# *MYC* or *BCL2* copy number aberration is a strong predictor of outcome in patients with diffuse large B-cell lymphoma

**DOI:** 10.18632/oncotarget.4073

**Published:** 2015-05-29

**Authors:** Ting-Xun Lu, Lei Fan, Li Wang, Jia-Zhu Wu, Kou-Rong Miao, Jin-Hua Liang, Qi-Xing Gong, Zhen Wang, Ken H. Young, Wei Xu, Zhi-Hong Zhang, Jian-Yong Li

**Affiliations:** ^1^ Department of Hematology, the First Affiliated Hospital of Nanjing Medical University, Jiangsu Province Hospital, Nanjing, China; ^2^ Department of Pathology, the First Affiliated Hospital of Nanjing Medical University, Jiangsu Province Hospital, Nanjing, China; ^3^ Department of Hematopathology, The University of Texas MD Anderson Cancer Center, Houston, TX, USA; ^4^ Collaborative Innovation Center for Cancer Personalized Medicine, Nanjing Medical University, Nanjing, China

**Keywords:** *MYC*, *BCL2*, copy number aberration, diffuse large B-cell lymphoma, pathology

## Abstract

Diffuse large B-cell lymphoma (DLBCL) is the most common non-Hodgkin lymphoma (NHL). Patients with DLBCL harboring *MYC* aberrations concurrent with *BCL2* or/and *BCL6* aberrations constitute a specific group with extremely poor outcome. In this study, we retrospectively investigated the incidence and prognosis of *MYC*, *BCL2*, and *BCL6* aberrations with DLBCL patients in Chinese population. We applied fluorescence *in situ* hybridization and immunohistochemical analysis in 246 DLBCL patients. The results showed that patients with *MYC* or *BCL2* copy number aberration (CNA) had significantly worse overall survival (OS) and progression-free survival (PFS) than negative cases (*P* < 0.0001). Patients with both *MYC* and *BCL2* CNA had similar outcomes to those with classic double hit lymphoma or protein double expression lymphoma (MYC and BCL2/BCL6 coexpression). By multivariate analysis, *MYC* CNA, *BCL2* CNA and double CNA were the independent worse prognostic factors. In conclusions, patients with *MYC* or *BCL2* CNA constituted a unique group with extremely poor outcome and may require more aggressive treatment regimens.

## INTRODUCTION

Diffuse large B-cell lymphoma (DLBCL), which is the most common group of non-Hodgkin lymphomas (NHL), accounts for 30%–40% of all lymphomas. The International Prognostic Index (IPI) is a clinical score that segregates DLBCL patients into four prognostic groups with distinct survival durations [1]. However, the differences in clinical features and treatment responses can also be affected by genetic and molecular features [2]. Genetic alterations can be of pivotal importance in establishing the correct diagnosis and predicting the course of disease [3].

*MYC* translocation, a biological hallmark of Burkitt lymphoma, can also be detected in DLBCL and B cell lymphoma unclassifiable with features intermediate between DLBCL and Burkitt lymphoma [4–6]. The t(14;18) translocation juxtaposes *BCL2* to the immunoglobulin heavy chain gene (*IGH*) enhancer, resulting in BCL2 protein overexpression and inhibition of apoptosis [7]. This translocation is found in 80%–90% of follicular lymphoma and 20%–30% of de novo DLBCL cases [8]. It was reported that *MYC* concurrent with *BCL2* or/and *BCL6* translocations in DLBCL, called double-hit lymphoma or triple-hit lymphoma (DHL/THL), determines highly aggressive clinical behavior with extremely poor outcome and resistance to chemotherapy [9–11]. In addition, protein expression (such as MYC and BCL2 or BCL6) also had important prognostic value with or without gene aberrations [12–16]. However, little attention has been paid to copy number aberration (CNA) of genes associated with DHL. Therefore, we investigated the incidence and prognosis of *MYC* and *BCL2* CNA in a population based study. Importantly, we compared the prognostic differences of double CNA with classic DHL and protein double expression (MYC and BCL2/BCL6 coexpression) lymphoma (DEL) and indicated the special value of double CNA which might be an important supplement to the DHL system.

## RESULTS

### The incidence of CNA, gene rearrangement and protein expression

Among the 246 DLBCL patients diagnosed in the First Affiliated Hospital of Nanjing Medical University, Jiangsu Province Hospital, fluorescence *in situ* hybridization (FISH) analysis was successfully performed in 240 cases and immunohistochemistry (IHC) analysis in 246 cases. Among 240 cases analyzed by FISH, *MYC* CNA was detected in 18 cases (7.5%), which was less frequent than *MYC* rearrangement {13.7% [33/240] cases had *MYC* translocation. Among these, 36.4% [12/33] cases accompanied *IG* [50% (6/12) each for *IGH* and *IGL*]}. *BCL2* CNA was observed in 65 (27.1%) cases, which was more common than *IGH*/*BCL2* rearrangement (12.5%, 30/240). In the 240 patients, 9 cases (3.8%) were identified as having CNA of both *MYC* and *BCL2* (double CNA), which was a little more frequent than classic DHL (2.9%, 7/240) in this study.

At the protein level, the incidence of MYC, BCL2, and BCL6 expression was 36.6% (90/246), 57.3% (141/246), and 65.9% (162/246), respectively. Among these, 26.0% (64/246) showed coexpression of MYC and BCL2, and 22.8% (56/246) showed coexpression of MYC and BCL6.

The distribution of CNA, rearrangement and expression of *MYC* (Table 1a) and *BCL2* (Table 1b) was showed in Table 1. Both *MYC* CNA (gain: *r* = 0.208, *P* = 0.002; amplification : *r* = 0.083, *P* = 0.340; gain plus amplification: *r* = 0.213, *P* = 0.001) and *MYC* rearrangement (*r* = 0.253, *P* < 0.001) were associated with MYC expression. A trend of association were observed between *MYC* CNA (gain plus amplification: *r* = 0.116, *P* = 0.082) and *MYC* rearrangement. *BCL2* CNA (gain: *r* = 0.397, *P* < 0.0001; amplification: *r* = 0.154, *P* = 0.029; gain plus amplification: *r* = 0.358, *P* < 0.0001) but not *BCL2* rearrangement (*r* = 0.124, *P* = 0.055) was associated with BCL2 expression. No association was found between *BCL2* CNA and *BCL2* rearrangement (*r* = 0.004, *P* = 0.956).

**Table 1a:** The distribution of *MYC* CNA, *MYC* rearrangement and MYC expression

Number of variables		*MYC*-R^+^	*MYC*-R^−^	MYC^+^	MYC^−^
*MYC* CNA^+^	gain	4	9	10	3
	amplification	1	4	3	2
*MYC* CNA^−^		28	194	74	148
*MYC* CNA*				3	3
*MYC*-R^+^				22	11
*MYC*-R^−^				65	142
*MYC*-R*				3	3

Abbreviation: R: rearrangement; CNA: copy number aberration; MYC^+^: MYC expression; ^+^: positive; *: no results.

**Table 1b:** The distribution of *BCL2* CNA, *BCL2* rearrangement and BCL2 expression

Number of variables		*BCL2*-R^+^	*BCL2*-R^−^	BCL2^+^	BCL2^−^
*BCL2* CNA^+^	gain	6	40	38	1
	amplification	2	17	18	8
*BCL2* CNA^−^		22	153	81	94
*BCL2* CNA^*^				4	2
*BCL2*-R^+^				22	8
*BCL2*-R^−^				115	95
*BCL2*-R*				4	2

Abbreviations: R: rearrangement; CNA: copy number aberration; BCL2^+^: BCL2 expression; ^+^: positive; *: no results.

### The association between gene CNA and clinical characteristics

Clinical characteristics analyzed for patients with gene CNA included age, sex, clinical stages, serum lactate dehydrogenase (LDH) level, performance status of Eastern Cooperative Oncology Group (ECOG PS), sites of extranodal involvement, IPI, B symptoms and cell of origin (COO). (Table 2). *MYC* CNA was associated with older age (*P* = 0.047) and higher IPI score (> 2) (*P* = 0.028). *BCL2* CNA was associated with older age (*P* = 0.005), poorer ECOG PS (≥ 2) (*P* = 0.016) and non-GCB preference (*P* = 0.005). Double *MYC* and *BCL2* CNA (MC+BC+) was associated with older age (*P* = 0.010).

**Table 2:** Clinical features with *MYC*, *BCL2* and double CNA

Characteristics	*MYC* CNA^+^	*MYC* CNA^−^	*P* value	*BCL2* CNA^+^	*BCL2* CNA^−^	*P* value	MC^+^BC^+^	MC^−^BC^−^	*P* value
	No. of cases (%)			No. of cases (%)			No. of cases (%)		
**Age (years)**	18	222		65	175		9	166	
≤ 60	7 (38.9)	139 (62.6)	0.047	30 (46.2)	116 (66.3)	0.005	2 (22.2)	111 (66.9)	0.010
> 60	11 (61.1)	83 (37.3)		35 (53.8)	59 (33.7)		7 (77.8)	55 (33.1)	
**Sex**	18	222		65	175		9	166	
Male	14 (77.8)	134 (60.4)	0.144	46 (70.8)	102 (58.3)	0.077	6 (66.7)	94 (56.6)	0.734
Female	4 (22.2)	88 (39.6)		19 (29.2)	73 (41.7)		3 (33.3)	72 (43.4)	
**Stage**	18	213		63	168		9	159	
III–IV	12 (66.7)	110 (51.6)	0.220	30 (47.6)	92 (54.8)	0.333	4 (44.4)	84 (52.8)	0.738
I–II	6 (33.3)	103 (48.4)		33 (52.4)	76 (45.2)		5 (55.6)	75 (47.2)	
**LDH**	18	213		63	168		9	159	
Elevated	9 (50.0)	90 (42.3)	0.524	32 (50.8)	67 (39.9)	0.136	4 (44.4)	62 (39.0)	0.739
Normal	9 (50.0)	123 (57.7)		31 (49.2)	101 (60.1)		5 (55.6)	97 (61.0)	
**ECOG PS**	18	222		65	175		9	166	
≥ 2	4 (22.2)	36 (16.2)	0.512	17 (26.2)	23 (13.1)	0.016	2 (22.2)	21 (12.7)	0.336
< 2	14 (77.8)	186 (83.8)		48 (73.8)	152 (86.9)		7 (77.8)	145 (87.3)	
**Extranodal involvement**	18	213		63	170		9	159	
≥ 2	4 (22.2)	44 (20.7)	0.772	15 (23.8)	35 (25.6)	0.595	2 (22.2)	31 (19.5)	1.000
< 2	14 (77.8)	169 (79.3)		48 (76.2)	135 (79.4)		7 (77.8)	128 (80.5)	
**IPI**	18	213		63	168		9	159	
3–5	9 (50.0)	53 (24.9)	0.028	19 (30.2)	43 (22.6)	0.486	4 (44.4)	38 (23.9)	0.230
0–2	9 (50.0)	160 (75.1)		44 (69.8)	125 (74.4)		5 (55.6)	121 (76.1)	
**B symptoms**	18	222		65	175		9	166	
Positive	5 (27.8)	79 (35.6)	0.504	27 (41.5)	57 (32.6)	0.196	3 (33.3)	55 (33.1)	1.000
Negative	13 (72.2)	143 (64.4)		38 (58.5)	118 (67.4)		6 (66.7)	111 (66.9)	
**COO (Hans)**	18	222		65	175		9	166	
GCB	5 (27.8)	93 (41.9)	0.241	17 (26.2)	81 (46.3)	0.005	2 (22.2)	78 (47.0)	0.183
Non-GCB	13 (72.2)	129 (58.0)		48 (73.8)	94 (53.7)		7 (77.8)	88 (53.0)	

Abbreviations: CNA: copy number aberration; MC^+^BC^+^: *MYC* CNA concurrent with *BCL2* CNA; MC^−^BC^−^: negative for both *MYC* CNA and *BCL2* CNA; COO: cell of origin; ECOG PS: performance status of Eastern Cooperative Oncology Group; GCB: germinal-center B-cell type; IPI: International Prognostic Index; LDH: lactate dehydrogenase.

### Survival analysis

#### Patients characteristics

In the present era of rituximab, we carried out subset analysis of 141 patients who treated with R-CHOP-like therapies. The median follow-up time was 30 months (3–112 months). The clinical features of the patients were listed in Table 3.

**Table 3:** The clinical features of the 141 patients who treated with R-CHOP-like therapies

Characteristics	No. of cases (%)
Age (years)	
≤ 60	82 (58.2)
Male	92 (65.2)
Stage III–IV	75 (53.2)
Elevated LDH	58 (41.1)
ECOG PS ≥ 2	29 (20.6)
Extranodal sites ≥ 2	34 (24.1)
IPI score of 3–5	48 (34.0)
B symptoms	52 (36.9)
COO (Hans)	
GCB	63 (44.7)
Non-GCB	78 (55.3)
Treatment	
R-CHOP	72 (51.1)
R-DA-EPOCH	22 (15.6)
R-CHOP-like^ζ^	47 (33.3)
Prophylactic CNS treatment^§^	25 (17.7)
Radiation^¶^	12 (8.6)
Treatment response	
CR(u)	112 (79.4)
PR	14 (10.0)
SD/PD	15 (10.6)

ζ Cases who received multiple regimens because of the following events: disease progression, cardiotoxicity of doxorubicin, accompanied hemophagocytic syndrome and extremely poor ECOG PS. The R-CHOP-like regimens including R-CDOP, R-CEOP and R-mini-CHOP.

§ Cases of with an increased risk of CNS events (paranasal sinus, testicular, bone marrow involvement) received 4–8 cycles of intrathecal methotrexate and/or cytarabine during the course of treatment.

¶ Cases with localized lesion received radiotherapy alone or radioimmunotherapy.

Abbreviations: COO: cell of origin; CR(u): complete remission (unconfirmed); DLBCL: diffuse large B-cell lymphoma; ECOG PS: performance status of Eastern Cooperative Oncology Group; GCB: germinal-center B-cell type; IPI: International Prognostic Index; LDH: lactate dehydrogenase; PR: partial remission; SD/PD: stable disease/progression of disease.

### Prognosis of *MYC* or *BCL2* CNA

**Figure 1:**
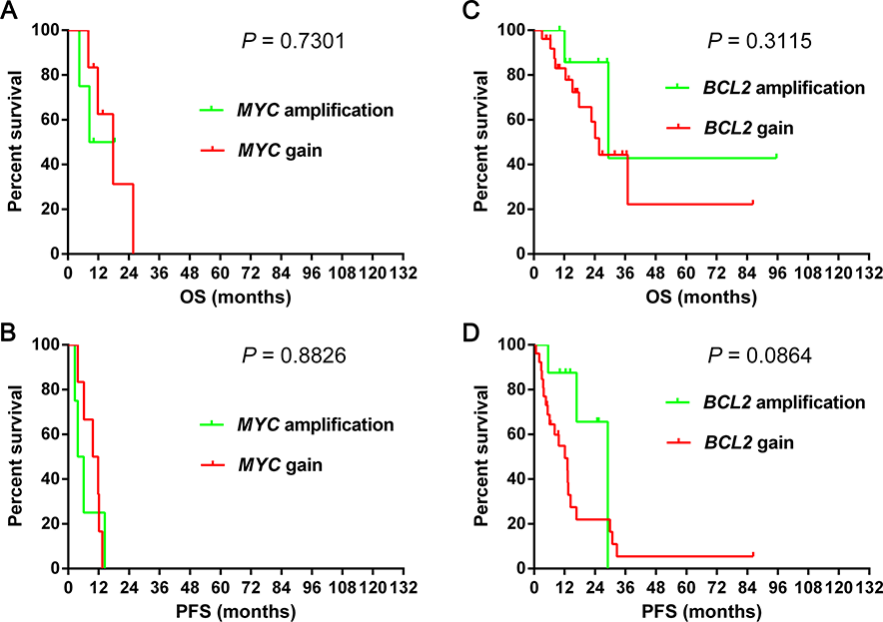
Overall survival and progression-free survival of cases grouped according to gain and amplification with *MYC* 1A–1B and *BCL2* 1C–1D in the R-CHOP-like group Abbreviations: OS: overall survival; PFS: progression-free survival.

**Figure 2:**
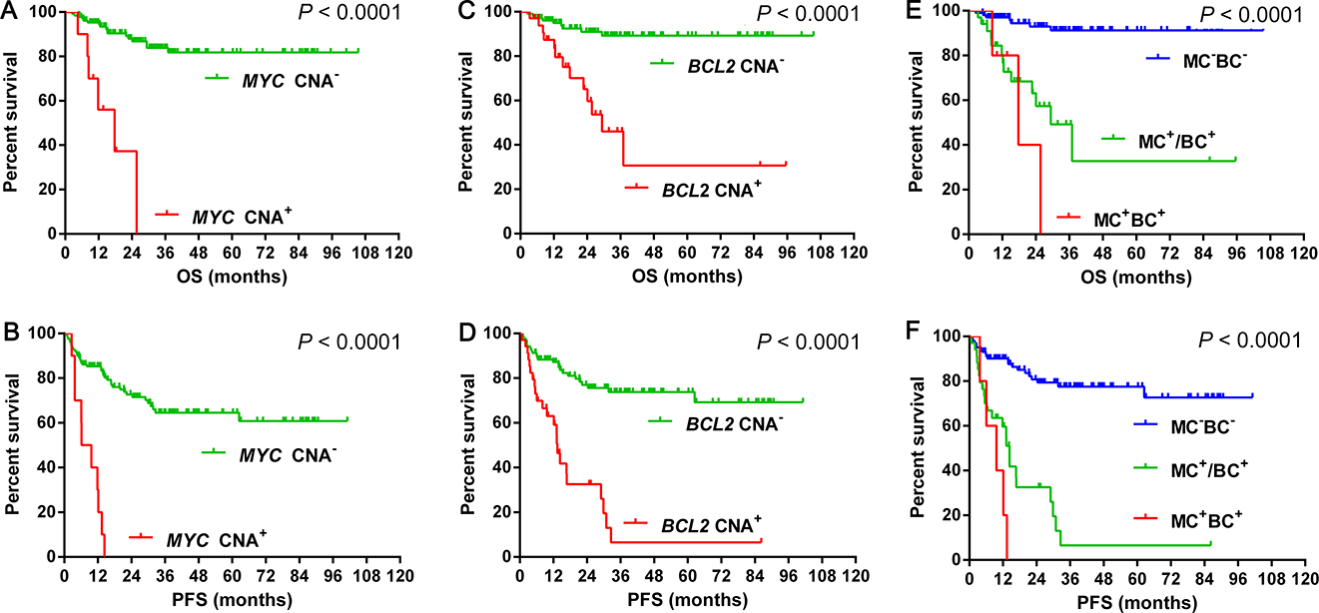
Overall survival and progression-free survival of cases grouped according to *MYC* CNA 2A–2B, *BCL2* CNA 2C–2D and double CNA 2E–2F Abbreviations: CNA: copy number aberration; OS: Overall survival; PFS: progression-free survival; MC: *MYC* CNA; BC: *BCL2* CNA; MC^+^BC^+^: *MYC* CNA concurrent with *BCL2* CNA; MC^+^/BC^+^: *MYC* CNA or *BCL2* CNA; MC^−^BC^−^: negative for both *MYC* CNA and *BCL2* CNA.

The incidences of *MYC* and *BCL2* CNA were 7.1% (10/141) and 24.1% (34/141), respectively. The median percentages of cells with *MYC* and *BCL2* CNA were 4% (2%–60%) and 5% (1%–80%), respectively. We then divided the *MYC* or *BCL2* CNA patients into gain (3–4 copies) and amplification (≥ 5 copies), and no survival differences were observed between the two groups (Figure 1A–1D). The presence of *MYC* CNA was significantly associated with worse OS (median OS, 17.8 months vs not reached, *P* < 0.0001) (Figure 2A) and PFS (median PFS, 8.0 months vs not reached, *P* < 0.0001) (Figure 2B). The presence of *BCL2* CNA was significantly associated with worse OS (median OS, 29.4 months vs not reached, *P* < 0.0001) (Figure 2C) and PFS (median PFS, 13.4 months vs not reached, *P* < 0.0001) (Figure 2D). In the current study, the best percentages of cells with CNA that predict outcome were 10% for both *MYC* and *BCL2*. By multivariate analysis, *BCL2* CNA was an independent prognostic factor for both OS and PFS while *MYC* CNA was an independent prognostic factor for OS (Table 4).

**Table 4a:** Univariate and multivariate analysis with OS

Variates	Univariate analysis (OS)			Multivariate analysis (OS)		
	HR	95% CI	*P* value	HR	95% CI	*P* value
ENI	2.484	1.496–4.126	0.0004	2.988	1.396–6.399	**0.0048**
LDH	2.746	1.661–4.541	< 0.0001	1.502	0.777–2.903	0.2262
Stage	1.558	0.950–2.553	0.0787	0.674	0.325–1.399	0.2900
IPI	2.801	1.714–4.576	< 0.0001	0.809	0.343–1.908	0.6279
ECOG PS ≥ 2	2.552	1.486–4.383	0.0007	1.603	0.833–3.085	0.1580
MYC^+^	5.515	3.339–9.108	< 0.0001	3.127	1.649–5.929	**0.0005**
BCL2^+^	2.086	1.243–3.500	0.0054	0.934	0.465–1.875	0.8471
BCL6^+^	0.665	0.415–1.066	0.0905	0.792	0.434–1.443	0.4458
Non-GCB	0.640	0.387–1.059	0.0825	0.710	0.335–1.502	0.3705
*MYC*-R^+^	4.715	2.873–7.739	< 0.0001	7.527	2.358–24.031	**0.0007**
DHL	1.724	1.116–2.563	0.0070	1.595	1.005–2.534	**0.0478**
*MYC* CNA^+^	5.344	2.790–10.234	< 0.0001	3.058	1.227–7.620	**0.0164**
*BCL2* CNA^+^	3.808	2.373–6.111	< 0.0001	2.619	1.417–4.840	**0.0002**
MC^+^BC^+^	3.504	2.460–4.991	< 0.0001	2.414	1.510–3.860	**0.0021**

Abbreviations: OS: overall survival; HR: hazard; ENI: extranodal involvement; LDH: serum lactate dehydrogenase; IPI: International Prognostic Index; ECOG PS: performance status of Eastern Cooperative Oncology Group; +: positive; GCB: germinal-center B-cell; R: rearrangement; DHL: double hit lymphoma; CNA: copy number aberration; MC^+^BC^+^: double CNA of *MYC* and *BCL2*.

**Table 4b:** Univariate and multivariate analysis with PFS

Variates	Univariate analysis (PFS)			Multivariate analysis (PFS)		
	HR	95% CI	*P* value	HR	95% CI	*P* value
ENI	2.064	1.387–3.071	0.0004	2.193	1.263–3.807	**0.0053**
LDH	1.945	1.363–2.777	0.0002	1.199	0.765–1.879	0.4286
Stage	1.590	1.109–1.280	0.0116	1.161	0.727–1.852	0.5320
IPI	2.010	1.374–2.939	0.0003	0.632	0.339–1.177	0.1482
ECOG PS≥2	1.842	1.195–2.841	0.0057	1.415	0.869–2.304	0.1630
B symptoms	1.781	1.255–2.526	0.0012	1.230	0.788–1.922	0.3618
MYC^+^	3.411	2.401–4.845	<0.0001	2.287	1.505–3.474	**0.0001**
BCL2^+^	1.789	1.237–2.587	0.0020	1.027	0.666–1.583	0.9048
Non-GCB	0.692	0.481–0.997	0.0479	0643	0.405–1.020	0.0607
*MYC*-R^+^	3.205	2.143–4.793	<0.0001	2.528	1.508–4.236	**0.0004**
DHL	1.610	1.180–2.196	0.0027	1.466	0.999–2.151	0.0504
*MYC* CNA^+^	3.521	2.058–6.022	<0.0001	1.610	0.873–2.969	0.1271
*BCL2* CNA^+^	2.827	1.972–4.052	<0.0001	2.066	1.349–3.165	**0.0008**
MC^+^BC^+^	2.613	1.981–3.446	<0.0001	2.067	1.341–3.186	**0.0010**

Abbreviations: PFS: progression-free survival; HR: hazard; ENI: extranodal involvement; LDH: serum lactate dehydrogenase; IPI: International Prognostic Index; ECOG PS: performance status of Eastern Cooperative Oncology Group; +: positive; GCB: germinal-center B-cell; R: rearrangement; DHL: double hit lymphoma; CNA: copy number aberration; MC^+^BC^+^: double CNA of *MYC* and *BCL2*.

### Prognosis of combining *MYC* CNA (MC) and *BCL2* CNA (BC)

The incidence of double CNA was 3.5%(5/141) in the R-CHOP-like group. The presence of double CNA was associated with worse OS (median OS, MC^+^BC^+^ vs. MC^+^/BC^+^: 17.8 vs 29.4 months, *P* = 0.307; MC^+^BC^+^ vs. MC^−^BC^−^: 17.8 months vs. not reached, *P* < 0.0001; MC^+^/BC^+^ vs. MC^−^BC^−^: 29.4 months vs. not reached, *P* < 0.0001) (Figure 2E) and PFS (median PFS, MC^+^BC^+^ vs. MC^+^/BC^+^: 9.7 vs. 14.4 months, *P* = 0.096; MC^+^BC^+^ vs. MC^−^BC^−^: 9.7 months vs. not reached, *P* < 0.0001; MC^+^/BC^+^ vs. MC^−^BC^−^: 14.4 months vs. not reached, *P* < 0.0001) (Figure 2F). By multivariate analysis, MCBC was an independent prognostic factor for both OS and PFS (Table 4).

### Survival differences with *MYC* CNA, *MYC* rearrangement and MYC expression

**Figure 3:**
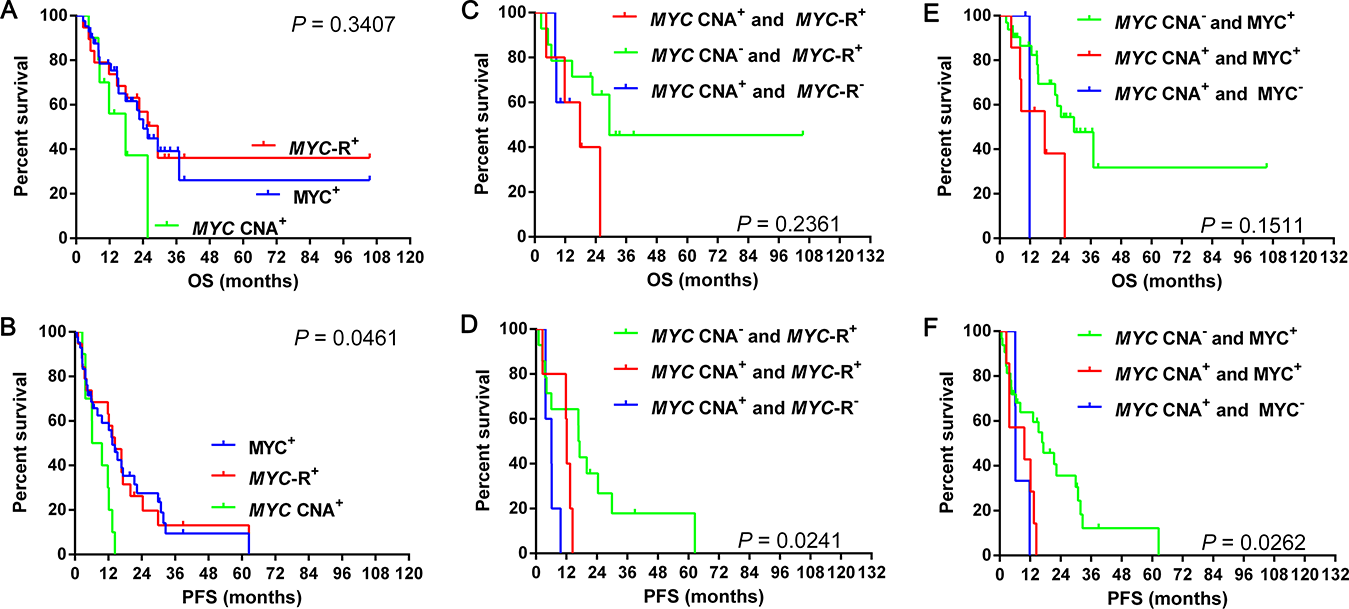
Overall survival and progression-free survival in cases grouped according to *MYC* CNA, *MYC* gene rearrangement and MYC expression 3A–3B Overall survival and progression-free survival in cases grouped according to MYC CNA or rearrangement alone and *MYC* CNA along with rearrangement **3C–3D** and *MYC* CNA or protein expression alone and *MYC* CNA along with protein expression **3E–3F**. Abbreviations: CNA: copy number aberration; R: rearrangement; OS: overall survival; PFS: progression-free survival.

The incidences of *MYC* rearrangement and MYC expression were 13.5% (19/141) and 29.8% (42/141), respectively. Patients with *MYC* CNA had similar OS (median OS: *MYC* CNA vs. *MYC* rearrangement: 17.8 vs. 29.4 months; *P* = 0.177; *MYC* CNA vs. MYC expression: 17.8 vs. 24.1 months, *P* = 0.180; *MYC* rearrangement vs. MYC expression: 29.4 vs. 24.1 months, *P* = 0.910) (Figure 3A) to cases with *MYC* rearrangement or MYC expression. Patients with *MYC* CNA tended to have a worse PFS (median PFS: *MYC* CNA vs. *MYC* rearrangement: 8.0 vs. 14.4 months; *P* = 0.019; *MYC* CNA vs. MYC expression: 8.0 vs. 13.4 months, *P* = 0.023; *MYC* rearrangement vs. MYC expression: 14.4 vs. 13.4 months; *P* = 0.972) (Figure 3B) than cases with *MYC* rearrangement or MYC expression.

Besides, we analyzed the survival differences among cases of *MYC* CNA, *MYC* rearrangement alone and *MYC* CNA along with rearrangement. Similar OS (median OS: *MYC* CNA alone vs. *MYC* rearrangement alone vs. *MYC* CNA along with rearrangement: not reached vs. 29.4 vs. 17.8 months, *P* = 0.236) were recognized among the three groups (Figure 3C, Table 5a). However, patients with *MYC* rearrangement alone tended to have longer PFS than cases with *MYC* CNA alone (median PFS: 17.0 vs. 6.1 months, *P* = 0.054) (Figure 3D, Table 5a) and *MYC* CNA along with rearrangement (median PFS: 17.0 vs. 12.2 months, *P* = 0.062) (Figure 3D, Table 5a). Patients with *MYC* CNA alone had worse PFS (median PFS: 6.1 vs. 12.2 months, *P* = 0.034) than cases with *MYC* CNA along with rearrangement (Figure 3D, Table 5a). We then analyzed the survival differences among cases of *MYC* CNA, MYC protein expression alone and *MYC* CNA along with protein expression. Patients with *MYC* CNA along with MYC expression tended to have shorter OS (median OS: *MYC* CNA along with MYC expression vs. MYC expression alone: 17.8 vs. 29.4 months, *P* = 0.060; *MYC* CNA along with MYC expression vs. *MYC* CNA alone: 17.8 vs. 11.8 months, *P* = 0.819) than cases with MYC expression alone but not *MYC* CNA alone (Figure 3E–3F, Table 5b). Patients with *MYC* CNA along with MYC expression had significantly shorter PFS (median PFS: *MYC* CNA along with MYC expression vs. MYC expression alone: 9.7 vs. 17.2 months, *P* = 0.018; *MYC* CNA along with MYC expression vs. *MYC* CNA alone: 9.7 vs. 6.2 months, *P* = 0.490) than cases with MYC expression alone but not *MYC* CNA alone (Figure 3E–3F, Table 5b). Similar OS (median OS: 11.8 vs. 29.4 months, *P* = 0.364) and PFS (median PFS: 6.2 vs. 17.2 months, *P* = 0.108) were observed between patients with *MYC* CNA alone and MYC expression alone (Figure 3E–3F, Table 5b).

**Table 5a:** The survival differences among *MYC* CNA^+^ alone, *MYC* rearrangement alone and *MYC* CNA^+^ along with *MYC* rearrangement

Survival	*MYC* CNA^+^ and *MYC*-R^+^		*MYC*-R^+^ alone		*MYC* CNA^+^ alone	
	χ^2^	*P* value	χ^2^	*P* value	χ^2^	*P* value
**Overall survival**						
*MYC* CNA^+^ and *MYC*-R^+^			2.819	0.093	0.066	0.797
*MYC*-R^+^ alone	2.819	0.093			0.339	0.560
*MYC* CNA^+^ alone	0.066	0.797	0.339	0.560		
**Progression-free survival**						
*MYC* CNA^+^ and *MYC*-R^+^			3.481	0.062	4.499	**0.034**
*MYC*-R^+^ alone	3.481	0.062			3.400	0.054
*MYC* CNA^+^ alone	4.499	**0.034**	3.400	0.054		

Abbreviations: CNA: copy number aberration; R: rearrangement; ^+^: positive.

**Table 5b:** The survival differences among *MYC* CNA^+^alone, MYC expression alone and *MYC* CNA^+^ along with MYC expression

Survival	*MYC* CNA^+^ and MYC+		MYC^+^ alone		*MYC* CNA^+^ alone	
	χ^2^	*P* value	χ^2^	*P* value	χ^2^	*P* value
**Overall survival**						
*MYC* CNA^+^ and MYC^+^			3.547	0.060	0.053	0.819
MYC^+^ alone	3.547	0.060			0.825	0.364
*MYC* CNA^+^ alone	0.053	0.819	0.825	0.364		
**Progression-free survival**						
*MYC* CNA^+^ and MYC^+^			5.608	**0.018**	0.477	0.490
MYC^+^ alone	5.608	**0.018**			2.585	0.108
*MYC* CNA^+^ alone	0.477	0.490	2.585	0.108		

Abbreviations: CNA: copy number aberration; MYC^+^: MYC expression; ^+^: positive.

### Survival differences with *BCL2* CNA, *BCL2* rearrangement and BCL2 expression

**Figure 4:**
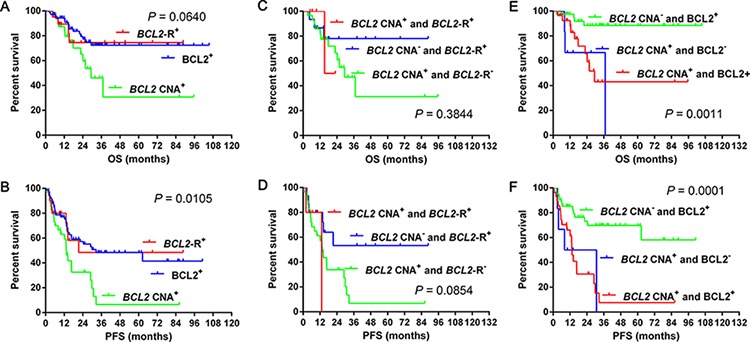
Overall survival and progression-free survival in cases grouped according to *BCL2* CNA, *BCL2* gene rearrangement and *BCL2* expression 4A–4B Overall survival and progression-free survival in cases grouped according to *BCL2* CNA or rearrangement alone and *BCL2* CNA along with rearrangement **4C–4D** and *BCL2* CNA or protein expression alone and *BCL2* CNA along with protein expression **4E–4F**. Abbreviations: CNA: copy number aberration; R: rearrangement; OS: overall survival; PFS: progression-free survival.

The incidences of *BCL2* rearrangement and BCL2 expression were 14.2% (20/141) and 51.1% (72/141), respectively. Patients with *BCL2* CNA showed decreased OS (median OS: *BCL2* CNA vs. *BCL2* rearrangement: 29.4 months vs. not reached; *P* = 0.204; *BCL2* CNA vs. BCL2 expression: 29.4 months vs. not reached, *P* = 0.019; *BCL2* rearrangement vs. BCL2 expression: both not reached, *P* = 0.764) (Figure 4A) and PFS (median PFS: *BCL2* CNA vs. *BCL2* rearrangement: 13.4 vs. 21.4 months; *P* = 0.048; *BCL2* CNA vs. BCL2 expression: 13.4 vs. 32.6 months, *P* = 0.003; *BCL2* rearrangement vs. BCL2 expression: 21.4 vs. 32.6 months; *P* = 0.958) (Figure 4B) than cases with *BCL2* rearrangement or BCL2 expression.

Meanwhile, we analyzed the survival differences among cases of *BCL2* CNA, *BCL2* rearrangement alone and *BCL2* CNA along with rearrangement. Similar OS was observed among above three groups (median OS: *BCL2* CNA alone vs. *BCL2* rearrangement alone vs. *BCL2* CNA along with rearrangement: 29.4 months vs. not reached vs. 19.1 months, *P* = 0.384) (Figure 4C, Table 6a). Patients with *BCL2* CNA alone had worse PFS than cases with *BCL2* rearrangement alone (median PFS: *BCL2* CNA alone vs. *BCL2* rearrangement alone: 13.4 months vs. not reached, *P* = 0.032) while patients with *BCL2* CNA along with rearrangement showed similar PFS to cases with *BCL2* CNA (median PFS: 13.2 vs. 13.4 months, *P* = 0.753) or *BCL2* rearrangement alone (median PFS: 13.2 months vs. not reached, *P* = 0.265) (Figure 4D, Table 6a). We then analyzed the survival differences among cases of *BCL2* CNA, protein expression alone and *BCL2* CNA along with protein expression. Patients with *BCL2* CNA along with BCL2 expression and *BCL2* CNA alone showed worse OS (median OS: *BCL2* CNA along with BCL2 expression vs. *BCL2* CNA alone vs. BCL2 expression alone: 29.4 vs 37.0 months vs. not reached, *P* = 0.002 for both) and PFS (median PFS: *BCL2* CNA along with BCL2 expression vs. BCL2 expression alone: 13.4 months vs. not reached, *P* < 0.001; *BCL2* CNA alone vs. BCL2 expression alone: 19.5 months vs. not reached, *P* = 0.011) than cases with BCL2 expression alone (Figure 4E–4F, Table 6b). Patients with *BCL2* CNA along with BCL2 expression had similar OS (median OS: 29.4 vs. 37.0 months, *P* = 0.587) and PFS (median PFS: 13.4 vs. 19.5 months, *P* = 0.899) to cases with *BCL2* CNA alone (Figure 4E–4F, Table 6b).

**Table 6a:** The survival differences among *BCL2* CNA^+^ alone, *BCL2* rearrangement alone and *BCL2* CNA^+^ along with *BCL2* rearrangement

Survival	*BCL2* CNA^+^ and *BCL2*-R^+^		*BCL2*-R^+^ alone		*BCL2* CNA^+^ alone	
	χ^2^	*P* value	χ^2^	*P* value	χ^2^	*P* value
**Overall survival**						
*BCL2* CNA^+^ and *BCL2*-R^+^			0.082	0.775	1.888	0.169
*BCL2*-R^+^ alone	0.082	0.775			0.015	0.901
*BCL2* CNA^+^ alone	0.015	0.901	1.888	0.169		
**Progression-free survival**						
*BCL2* CNA^+^ and *BCL2*-R^+^			1.245	0.265	0.099	0.753
*BCL2*-R^+^ alone	1.245	0.265			4.596	**0.032**
*BCL2* CNA^+^ alone	0.099	0.753	4.596	**0.032**		

Abbreviations: CNAs: copy number aberration; R: rearrangement; ^+^: positive.

**Table 6b:** The survival differences among *BCL2* CNA^+^ alone, BCL2 expression alone and *BCL2* CNA^+^ along wvith BCL2 expression

Survival	*BCL2* CNA^+^ and BCL2^+^		BCL2^+^ alone		*BCL2* CNA^+^ alone	
	χ^2^	*P* value	χ^2^	*P* value	χ^2^	*P* value
**Overall survival**						
*BCL2* CNA^+^ and BCL2^+^			9.847	**0.002**	0.295	0.587
BCL2^+^ alone	9.847	**0.002**			9.959	**0.002**
*BCL2* CNA ^+^alone	0.295	0.587	9.959	**0.002**		
**Progression-free survival**						
*BCL2* CNA^+^ and BCL2^+^			15.868	**< 0.001**	0.016	0.899
BCL2^+^ alone	15.868	**< 0.001**			6.505	**0.011**
*BCL2* CNA^+^ alone	0.016	0.899	6.505	**0.011**		

Abbreviations: CNA: copy number aberration; BCL2^+^: BCL2 expression; ^+^: positive.

### Survival differences with double CNA, classic DHL and DEL

**Figure 5:**
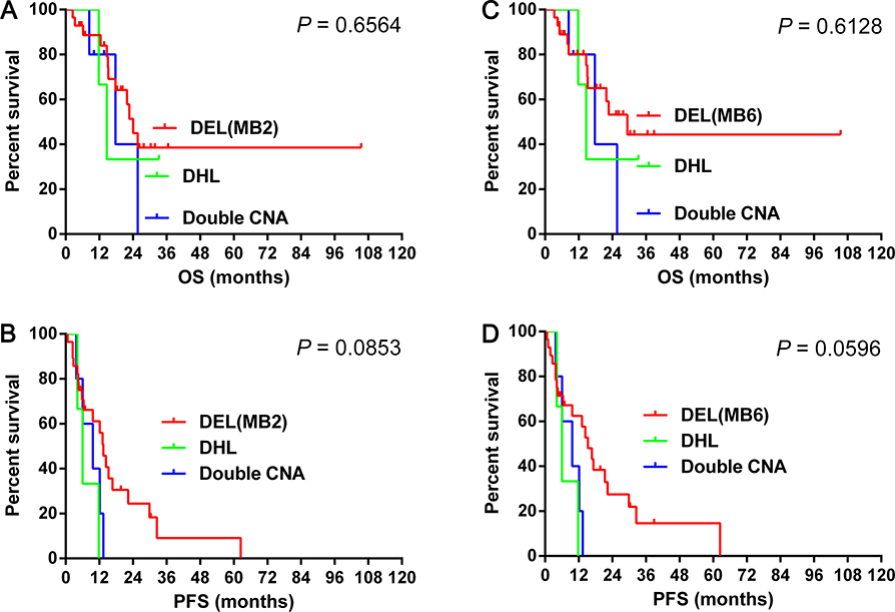
The differences of overall survival **5A–5B** and progression-free survival 5C–5D in cases grouped according to double CNA, classic DHL and DEL Abbreviations: CNA: copy number aberration; OS: overall survival; PFS: progression-free survival; DHL: double hit lymphoma; DEL: double expression lymphoma; MB2: double expression of MYC and BCL2 ; MB6: double expression of MYC and BCL6.

Double CNA had similar OS (median OS: double CNA vs. classic DHL: 17.8 vs. 14.7 months, *P* = 0.850; double CNA vs. DEL: 17.8 vs. 24.1 months, *P* = 0.425; classic DHL vs. DEL: 14.7 vs. 24.1 months, *P* = 0.571) (Figure 5A) and PFS (median PFS: double CNA vs. classic DHL: 9.7 vs. 6.0 months, *P* = 0.338; double CNA vs. DEL: 9.7 vs. 13.4 months, *P* = 0.127; classic DHL vs. DEL: 6.0 vs. 13.4 months, *P* = 0.086) (Figure 5B) to classic DHL or DEL (MYC and BCL2). Double CNA also had similar OS (median OS: double CNA vs. classic-DHL: 17.8 vs. 14.7 months, *P* = 0.850; double CNA vs. DEL: 17.8 vs. 29.4 months, *P* = 0.366; classic DHL vs. DEL: 14.7 vs. 29.4 months, *P* = 0.583) (Figure 5C) and PFS (median PFS: double CNA vs. classic DHL: 9.7 vs. 6.0 months, *P* = 0.338; double CNA vs. DEL: 9.7 vs. 15.3 months, *P* = 0.071; classic DHL vs. DEL: 6.0 vs. 15.3 months, *P* = 0.098) (Figure 5D) to classic DHL or DEL (MYC and BCL6).

## DISCUSSION

In this study, we performed a systematic investigation of the incidences and prognostic significances of *MYC* and *BCL2* aberrations of DLBCL in a Chinese population. As far as we know, this is the first report to compare the prognosis among CNA, rearrangement and protein expression of *MYC* or *BCL2*. The results showed *MYC* or *BCL2* CNA had more significant prognostic value than gene rearrangement or protein expression. It is noteworthy that we also compared the different prognostic values of double CNA with classic DHL and DEL. Double CNA had remarkable prognostic significance and might be an indispensable component of classic DHL.

The addition of rituximab to classic CHOP chemotherapy has significantly improved the outcome of DLBCL patients. However, more than one-third of the patients experience relapse and eventually die within 1–2 years [15]. It is necessary to identify such poor-risk patients who may benefit from alternative treatment strategies. Although gene expression profiling studies uncover biological heterogeneity with prognostic significance in DLBCL, the incorporation of this information into treatment algorithms awaits further investigation. This situation has motivated us to assess the clinical and prognostic significances of protein expression and rearrangement, such as *MYC*, *BCL2*, and *BCL6*, in a series of representative patients with DLBCL [17].

Recent studies have highlighted the importance of assessing *MYC* rearrangement in aggressive B-cell lymphomas, mainly DLBCL, as well as the detection of protein expression [3, 14, 18–20]. However, little knowledge has been investigated for CNA of genes associated with DHL, which might also be important for the prognosis of DLBCL. In the current study, we evaluated the clinical features, genetic characteristics, and prognostic significance of 246 cases of DLBCL with *MYC* or *BCL2* CNA, rearrangement and protein expression.

It is noteworthy that we observed a series of patients with additional copies of *MYC* (7.5%) or *BCL2* (27.1%), which were further confirmed by centromere 8 and *BCL2* break apart probes. The attribute of *MYC* or *BCL2* CNA, although not systematically investigated, has been reported in recent and previous studies [8, 16, 21, 22]. However, most studies did not analyze the prognostic significance of CNA [16, 21] or use a centromere reference and additional probe to distinguish polysomies or single gene break apart [8, 20]. Yoon *et al*. [22] previously observed increased copy number of *MYC* and *BCL2* in 7.1% and 11.7% of DLBCL patients, respectively, more frequently in the non-GCB subtype. Our study showed that *BCL2* but not *MYC* CNA was associated with the non-GCB subtype. However, the incidence of *BCL2* CNA detected in our study was higher than theirs, probably due to different ethnic background or geographic variation. Stasik *et al*. [23] recently described a colorimetric *in situ* hybridization (CISH) method for detecting extra copies of the *MYC* gene in DLBCL and frequent occurrence of excess copies of discrete *MYC* signals (38%) in the context of diploidy or polyploidy of chromosome 8, which was correlated with increased mRNA signals and poor outcome. Although CISH might be more accurate to study *MYC* gene, it is not widely used. The most common and classic method to analyze *MYC* gene aberration is still conventional FISH. Additionally, conventional FISH is much easier to perform routinely. We confirmed these results and found that *MYC* CNA was associated with poor outcome [24, 25]. Similarly, another report had also indicated adverse effects on survival of *MYC* or *BCL2* CNA [22]. However, they focused on *MYC* or *BCL2* CNA alone, and not in combination (double CNA), which predicted worse OS and PFS, similar to classic DHL.

As is reported that *MYC* rearrangement was associated with decreased OS and PFS, [19, 26–28] while *BCL2* rearrangement, in keeping with most reports [12, 29–31], was not predictive of both OS or PFS in patients treated with R-CHOP. Four recent studies evaluated the effect of MYC expression in DLBCL patients treated with R-CHOP [14, 15, 19, 27]. However, only one study, which was in line with ours, showed that MYC expression predicted poor survival [19]. The prognosis of BCL2 expression was more confusing in previous reports [16, 21]. Based on these results, we compared the differences among prognostic values of CNA, gene rearrangement and protein expression. Unexpectedly, patients with *BCL2* CNA showed much shorter OS and PFS than those with *BCL2* rearrangement or BCL2 expression. However, patients with *MYC* CNA showed similar OS and PFS to those with *MYC* rearrangement or MYC expression. These results indicated that CNA of *MYC* or *BCL2* had significant prognostic value and should not be neglected.

Similar to previous researches [15, 19, 20, 27], we confirmed that patients with MYC and BCL2 coexpression (DEL) had extremely poor OS and PFS. Besides, we found that patients with MYC and BCL6 double expression predicted inferior outcome as well. Accordingly, we then compared the survival differences among double CNA, classic DHL and DEL. Surprisingly, no significant difference of OS or PFS was recognized among them, which indicated that double CNA had similar prognostic value to classic DHL and DEL and should not be overlooked in future studies.

In summary, patients with *MYC* or *BCL2* CNA constituted another group of patients with extremely poor outcome. In multivariate analysis, *MYC* CNA (except for PFS), *BCL2* CNA and double CNA were independent prognostic factors. Though the limited cases analyzed in our study, as far as we know, it is the first study comparing the different prognostic values of *MYC* and *BCL2* at three distinct levels, which demonstrated the important prognosis of CNA. Thus, we suggest that patients with DLBCL harboring *MYC* or *BCL2* CNA constituted a unique group with extremely poor outcome and may require more aggressive treatment regimens.

## MATERIALS AND METHODS

### Patients

All patients enrolled informed consent in accordance with requirements of the Declaration of Helsinki, and the research project was approved by the University and Institutional Review Boards. We retrospectively enrolled 246 adult patients with de novo DLBCL that had been diagnosed between February 2006 and January 2014 in the First Affiliated Hospital of Nanjing Medical University, Jiangsu Province Hospital. All cases were diagnosed according to World Health Organization (WHO) classification criteria. Cases were excluded if patients had a history of low grade B-cell lymphoma, primary cutaneous DLBCL, primary DLBCL of the central nervous system, primary mediastinal B-cell lymphoma or AIDS/HIV infection.

### IHC

**Figure 6:**
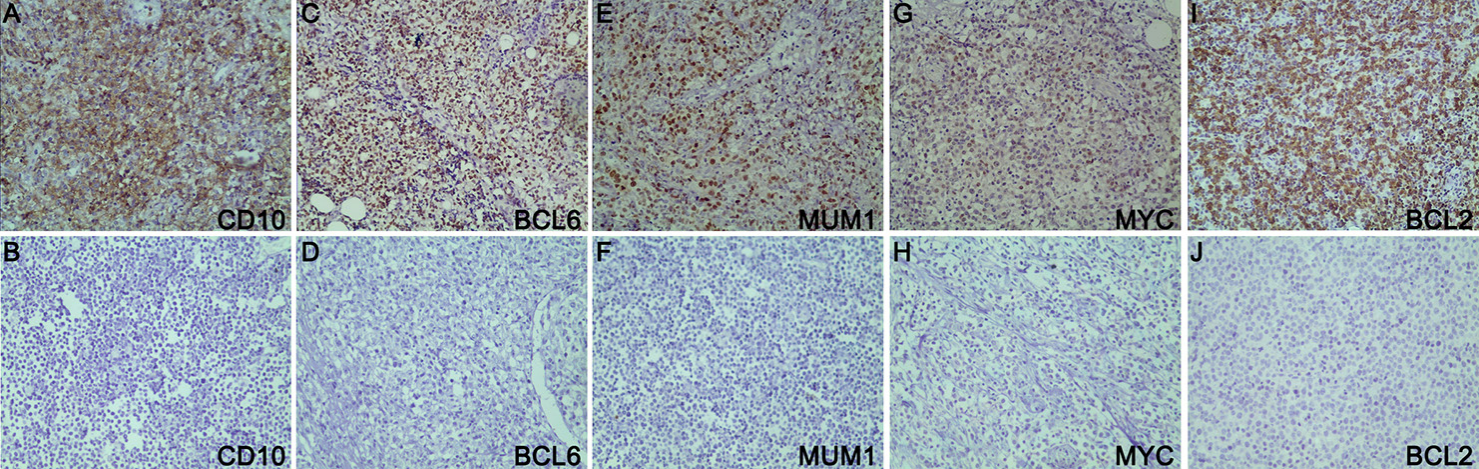
The results of immunohistochemistry The proteins applied in this study were CD10 **6A–6B,** BCL6 **6C–6D,** MUM1 **6E–6F**, MYC **6G–6H** and BCL2 **6I–6J.**

IHC (Figure 6) was performed on 4 μm sections with formalin-fixed paraffin-embedded (FFPE) specimens. Antibodies applied in the study including CD10 (clone 56C6; Dako, cut-off: 30%), MYC (clone Y69; Abcam, cut-off: 40%), BCL2 (clone 124; Dako, cut-off: 50%), BCL6 (clone LN22; Dako, cut-off: 30%), and MUM1 (clone MUM1p; Dako, cut-off: 30%). The COO was classified according to Hans algorithm [32].

### FISH

**Figure 7:**
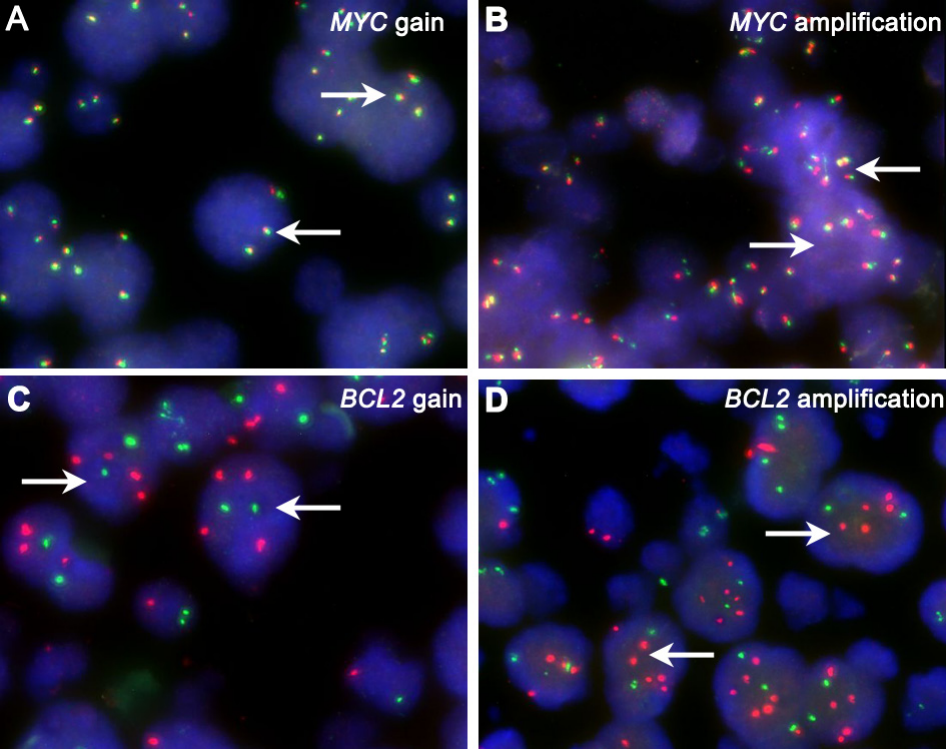
CNA of *MYC* and *BCL2* White arrows show *MYC* and *BCL2* gain **7A, 7C** and amplification **7B, 7D**. Abbreviation: CNA: copy number aberration.

FISH was carried out according to manufacturer's instructions on FFPE tissue sections with the following probes: *MYC* dual-color, break apart translocation probe (Vysis LSI) and *IGH/BCL2* dual-color, and dual fusion translocation probe (Vysis LSI). For cases with *MYC* translocation, the *IGH/MYC/CEP* 8 tri-color, dual fusion translocation probe (Vysis LSI), *BCL6* dual-color break apart rearrangement probe (Vysis LSI), *IG-kappa* (*IGK*) DNA FISH probe, Split Signal (code Y5416; Dako) and *IG-lambda* (*IGL*) DNA FISH probe, Split Signal (code Y5412; Dako) were applied to further analyze on *MYC* concurrent gene rearrangements and partner genes. Cases with three or more *BCL2* signals and normal *IGH* signals (*BCL2*/*IGH* probe) and without *BCL2* gene break apart (*BCL2* gene break apart probe) were considered as *BCL2* CNA [33]. Cases with three or more *MYC* signals (*IGH*/*MYC*/*CEP 8* tri-color, dual fusion translocation probe) along with two aqua signals of centromere 8 per nuclei were considered as *MYC* CNA [22]. Three or four copies of the gene studied was considered a gain, whereas more than four copies was considered as amplification (Figure 7) [34]. For probe signal scoring, a minimum of 200 interphase nuclei was examined. The cut-off levels for the probes were established by evaluating the split signal distribution in samples of reactive lymphoid tissues, calculating the mean number of split signals plus three times the standard deviation. The cut-off levels for positive values (mean of normal control ± 3 SD) were 14%, 5% and 7% for *MYC* break apart probe, *IGH*/*BCL2* dual-fusion probe and *BCL6* break apart probe, respectively.

### Statistical analyses

OS and PFS were defined according to Cheson 2014 [35]. Statistical analyses were performed with use of SPSS software, version 20.0. The Chi-squared and Fisher exact tests were used to determine differences in the frequencies between groups. The Spearman test was used to analyze correlations between different variables. Survival curves were plotted by using Kaplan-Meier method and were compared by using log-rank test. For all tests, a probability value of less than 0.05 (2-sided) was considered statistically significant.
